# Data on the densification during sintering of binder jet printed samples made from water- and gas-atomized alloy 625 powders

**DOI:** 10.1016/j.dib.2016.11.078

**Published:** 2016-11-28

**Authors:** Amir Mostafaei, Eamonn T. Hughes, Colleen Hilla, Erica L. Stevens, Markus Chmielus

**Affiliations:** Department of Mechanical Engineering and Materials Science, University of Pittsburgh, Pittsburgh, PA 15261, USA

**Keywords:** Additive manufacturing, Powder bed binder jet printing, Inconel 625, Sintering, Gas atomized, Water atomized, Powder analysis, Densification, Microstructure

## Abstract

Binder jet printing (BJP) is a metal additive manufacturing method that manufactures parts with complex geometry by depositing powder layer-by-layer, selectively joining particles in each layer with a polymeric binder and finally curing the binder. After the printing process, the parts still in the powder bed must be sintered to achieve full densification (A. Mostafaei, Y. Behnamian, Y.L. Krimer, E.L. Stevens, J.L. Luo, M. Chmielus, 2016; A. Mostafaei, E. Stevens, E. Hughes, S. Biery, C. Hilla, M. Chmielus, 2016; A. Mostafaei, Y. Behnamian, Y.L. Krimer, E.L. Stevens, J.L. Luo, M. Chmielus, 2016) [1–3]. The collected data presents the characterization of the as-received gas- and water-atomized alloy 625 powders, BJP processing parameters and density of the sintered samples. The effect of sintering temperatures on the microstructure and the relative density of binder jet printed parts made from differently atomized nickel-based superalloy 625 powders are briefly compared in this paper. Detailed data can be found in the original published papers by authors in (A. Mostafaei, J. Toman, E.L. Stevens, E.T. Hughes, Y.L. Krimer, M. Chmielus, 2017) [4].

**Specifications Table**

**Table:** 

Subject area	*Materials Science and Engineering*
More specific subject area	*Additive Manufacturing of nickel superalloy 625*
Type of data	*Figures*
How data was acquired	*Characterization of gas- and water-atomized powders and BJP sintered samples were conducted using scanning electron microscopy (SEM), micro-computed tomography (micro-CT), laser particle analysis and optical microscopy (OM).*
Data format	*Analyzed*
Experimental factors	*A powder bed binder jet printer (M-Flex ExOne) was utilized to produce alloy 625 parts made of two differently atomized powders, water-atomized (WA) and gas-atomized (GA), with the following printing parameters: layer height of 100 μm, recoat speed of 130 \ mm/s, oscillator speed of 2050 rpm, roller speed of 250 rpm, roller traverse speed of 15 mm/s, and drying speed of 17 mm/s*[1], [2], [3].
Experimental features	*After printing, BJP parts (“green parts”) were cured at 175 °C in a JPW Design & Manufacturing furnace and then sintered in a Lindberg tube furnace in an alumina powder bed under vacuum with the following heating profile: heating at 5 °C/min from RT to 600 °C, 3.2 °C/min to 1000 °C, 2.8 °C/min to the holding temperature (1225 °C, 1240 °C, 1255 °C, 1270 °C, 1285 °C, and 1300 °C), holding for 4 h and then cooling at 1 °C/min to 1200 °C, 3.1 °C/min to 500 °C and finally to RT with a temperature stability of 1 °C*[3].
Data source location	*University of Pittsburgh, Pittsburgh, Pennsylvania, United States*
Data accessibility	*Data is with the article*

**Value of the data**

• The presented printing parameters assist researchers in obtaining the highest green part density of binder jet printed samples of other Ni-based alloys.
• Data allows one to determine process-property relationships for binder jet printed parts as well as the effect of different atomization methods on the densification and morphology of the BJP sintered samples.
• A detailed data overview on the densification of BJP alloy 625 may help in designing the additive manufacturing process.

## 1 Data

**Fig. 1:**
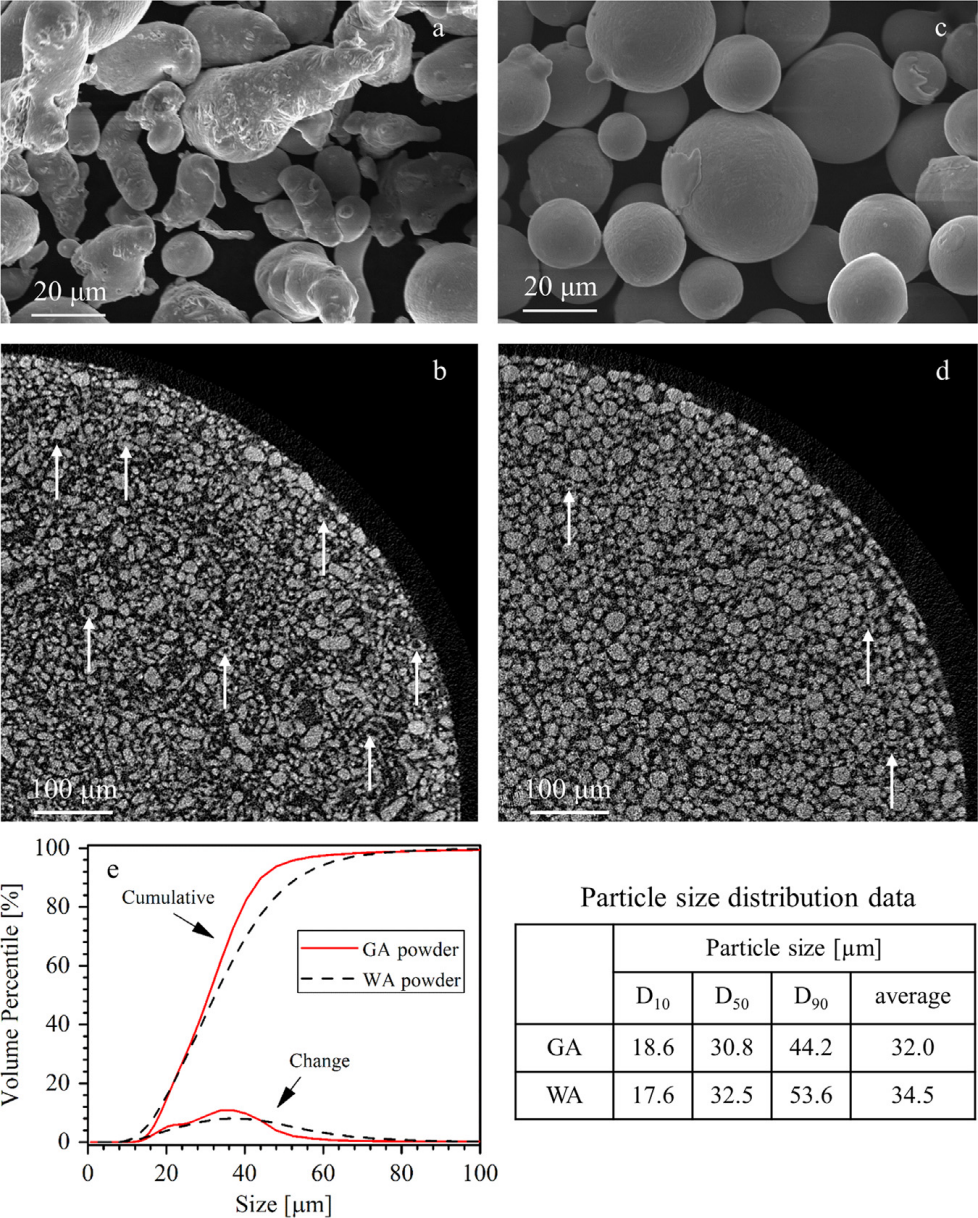
SEM and micro-CT micrographs taken of (a and b) WA and (c and d) GA powders, illustrating powder morphology, size distribution and internal porosity of powders. (e) Particle size distribution data was collected using a laser particle size analyzer.

**Fig. 2:**
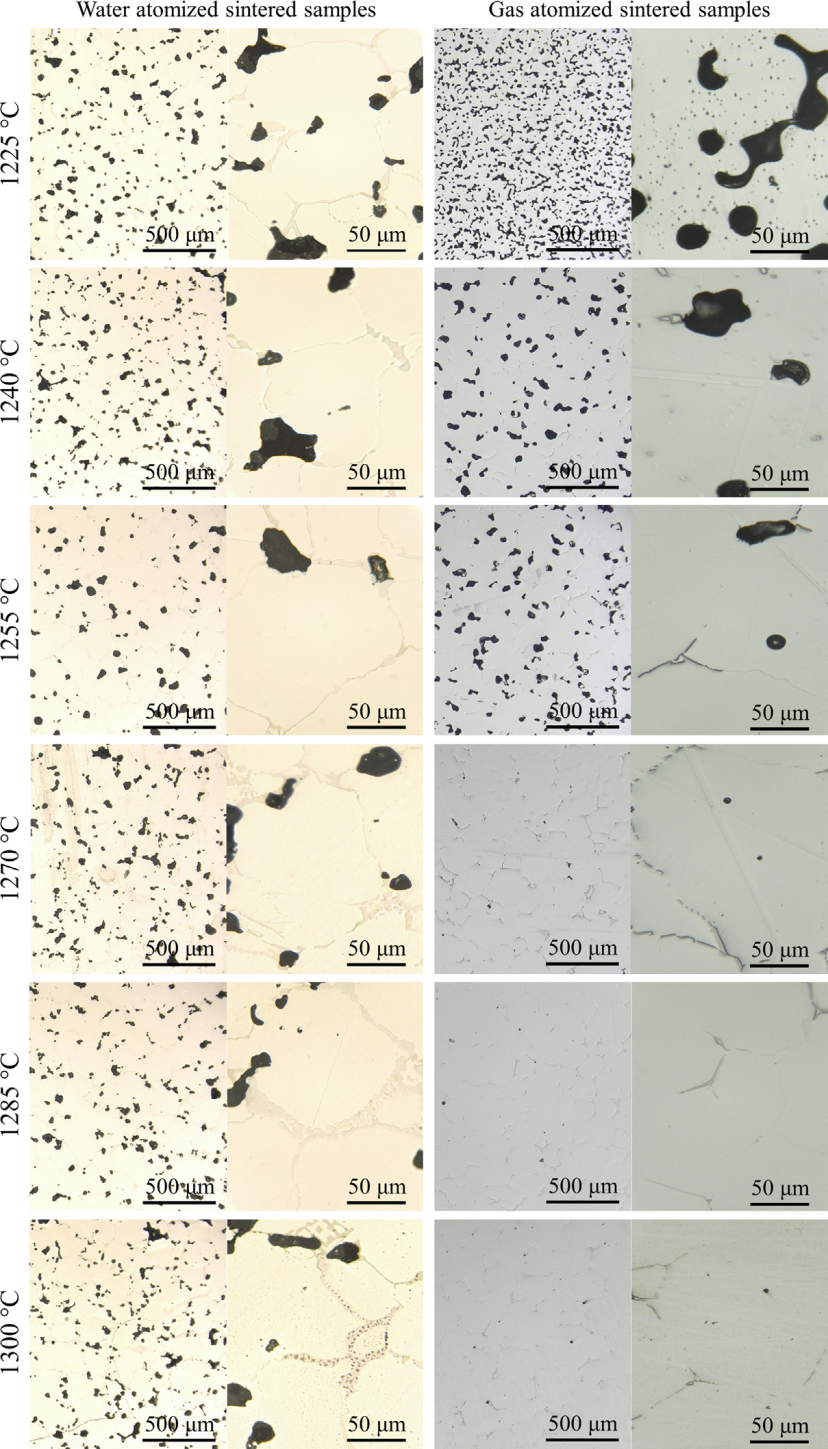
Optical microscopy micrographs taken from the WA and GA BJP samples sintered at different temperatures ranging from 1225 °C to 1300 °C.

**Fig. 3:**
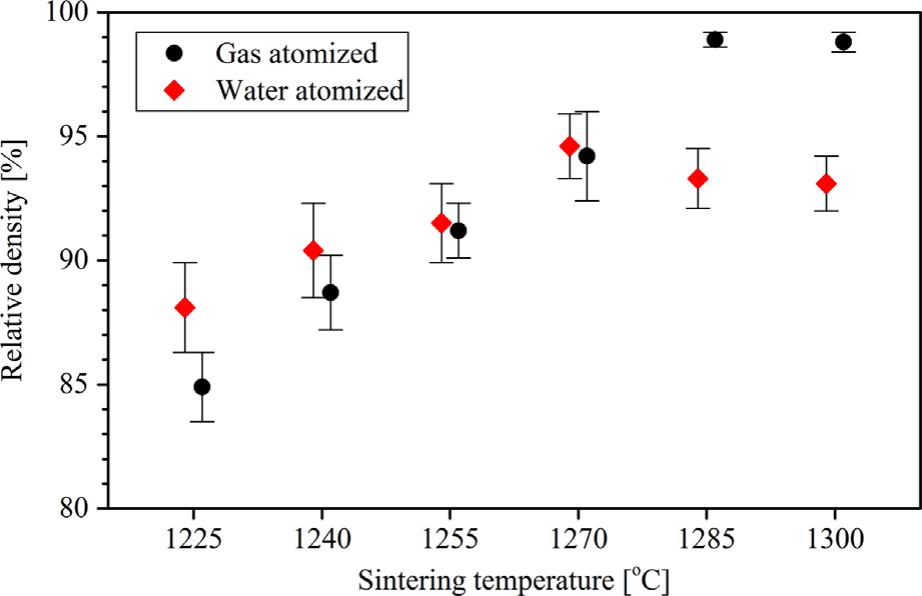
Relative density measurements obtained from image analysis of optical micrographs.

**Fig. 4:**
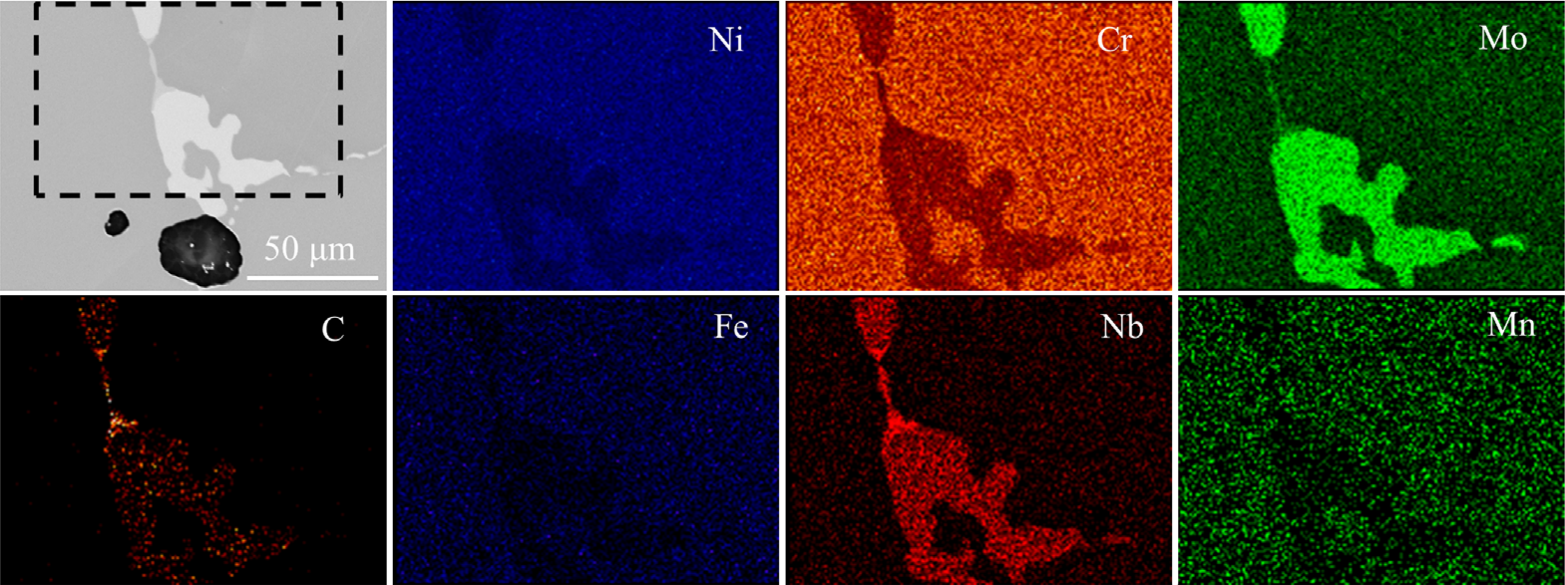
SEM and EDS mapping micrographs taken from the WA sample sintered at 1300 °C.

The data presented here can be divided into two parts: (1) characterization of the two atomized powders including gas- and water-atomized powders (Fig. 1, Fig. 2) densification observation of the BJP alloy 625 samples made from gas- and water-atomized powders in terms of optical microscopy micrographs (Fig. 2, Fig. 3, Fig. 4). The microscopy observations and density measurements conducted in this paper are based on experimental results presented in the publication from the authors [4].

## 2 Experimental design, materials and methods

Brief data overview of powder characterizations on the GA and WA powders are illustrated in Fig. 1. The data presented here includes powder size, shape, morphology and internal porosity collected using SEM, micro-CT and particle size distribution.

The WA alloy 625 powder (HAI Advanced Material Specialists, Inc.) was irregular in shape having been created via an air-melted water atomization method while the GA alloy 625 powder (Carpenter Technology Corporation) was spherical in shape having been created via an air-melted nitrogen atomization method. As shown in Fig. 1e, the GA powder had smaller particle size distribution ranging from 18.6 μm to 44.2 μm with the average particle size of 32 μm; however, the WA powder had wider particle size distribution between 17.6 μm and 53.6 μm with the average particle size of 34.5 μm.

Morphology as well as internal porosity of the WA and GA powders were observed with a Bruker SkyScan1272 micro-computed tomography scanner (micro-CT) at 100 kV and 100 μA and a 0.11 mm Cu filter, averaging of 10 frames, and angular range of 0°–180° with 0.2°–0.3° steps. Powder particles were filled into a low absorbance 1.5 mm plastic straw, gently compacted to reduce particle movement during scanning procedure and then scanned without random movement. It is found that WA powder had more internal porosity compared to GA powder.

To fabricate three-dimensional samples, an M-Flex ExOne printer was used to print small coupons with dimensions of 10 mm×10 mm×5 mm. BJP samples from the WA and GA powders were printed with the following printing parameters: recoat speed of 130 mm/s, oscillator speed of 2050 rpm, roller speed of 250 rpm, roller traverse speed of 15 mm/s, drying speed of 17 mm/s, and printing layer thickness of 100 μm [1−3]. The total number of printed layers was 50. A cleaner made of 2-butoxyethanol and a water-soluble binder made of ethylene glycol monomethyl ether and diethylene glycol were used in this research [4].

The microstructural evolutions of the BJP samples due to increasing sintering temperature from 1225 °C to 1300 °C are shown in Fig. 2. Sintered coupon samples were cut using a wire saw, mounted using epoxy and hardener, progressively ground up to grit-1200, polished to a final step of colloidal silica, and etched with a Kallings solution. We aimed to observe the effect of different sintering temperatures on the relative density, grain size, grain growth and pore size of the BJP sintered samples. Optical micrographs (Fig. 3) revealed that the maximum relative density of 95% and 99.2% were obtained at sintering temperatures of 1270 °C and 1285 °C for the WA and GA BJP samples, respectively. The micrographs (Fig. 3) also show increasing precipitation due to segregation of alloying elements inside the grains and/or at the grain boundaries for GA and WA samples as the sintering temperature was increased to 1300 °C. Fig. 4 illustrates precipitation at the grain boundary of the WA sample sintered at 1300 °C.

## References

[1] Mostafaei A., Behnamian Y., Krimer Y.L., Stevens E.L., Luo J.L., Chmielus M. (2016). Effect of solutionizing and aging on the microstructure and mechanical properties of powder bed binder jet printed nickel-based superalloy 625. Mater. Des..

[2] Mostafaei A., Stevens E., Hughes E., Biery S., Hilla C., Chmielus M. (2016). Powder bed binder jet printed alloy 625: densification, microstructure and mechanical properties. Mater. Des..

[3] Mostafaei A., Behnamian Y., Krimer Y.L., Stevens E.L., Luo J.L., Chmielus M. (2016). Brief data overview of differently heat treated binder jet printed samples made from argon atomized alloy 625 powder. Data Brief..

[4] Mostafaei A., Toman J., Stevens E.L., Hughes E.T., Krimer Y.L., Chmielus M. (2017). Microstructural evolution and mechanical properties of differently heat-treated binder jet printed samples from gas- and water-atomized alloy 625 powders. Acta Mater..

